# Efficacy and Safety of Paracetamol for Patent Ductus Arteriosus Closure in Preterm Infants: An Updated Systematic Review and Meta-Analysis

**DOI:** 10.3389/fped.2019.00568

**Published:** 2020-02-18

**Authors:** Yingqi Xiao, Hui Liu, Rujun Hu, Qiang You, Min Zeng, Xiaolian Jiang

**Affiliations:** ^1^West China School of Nursing/ West China Hospital, Sichuan University, Chengdu, China; ^2^Key Laboratory of Birth Deficits and Related Diseases of Women and Children, West China Second University Hospital, Sichuan University, Chengdu, China; ^3^Department of Pharmacy, Chengdu University of Traditional Chinese Medicine, Chengdu, China; ^4^Department of Pharmacy, West China Second University Hospital, Sichuan University, Chengdu, China

**Keywords:** ductus arteriosus, patent, infant, premature, paracetamol, ibuprofen, indomethacin

## Abstract

**Background:** Indomethacin and ibuprofen, two commonly used prostaglandin inhibitors, are the drugs of choice for patent ductus arteriosus. However, paracetamol is an alternative choice when these drugs are ineffective or contraindicated. This study aimed to confirm paracetamol's efficacy and safety compared with those of other drugs or placebos for patent ductus arteriosus closure in premature infants.

**Methods:** We conducted a literature search using the Cochrane Library, PubMed, CINAHL, and EMBASE databases for randomized controlled trials and quasi-randomized controlled trials. We used the Preferred Reporting Items for Systematic Reviews and Meta-Analyses (PRISMA) guidelines to direct the process and PICO (P, population; I, intervention/interest; C, comparator; O, outcome) principle to constitute the theme. We combined the research data through qualitative summaries or meta-analyses.

**Results:** The final analyses included 15 trials (*N* = 1,313). No significant differences were noted between paracetamol and ibuprofen except for shorter mean days needed for patent ductus arteriosus closure, lower risk of gastrointestinal bleeding, and hyperbilirubinemia. No significant difference existed between paracetamol and indomethacin. Oral paracetamol was more effective than placebo in infants weighing 1,501–2,500 g.

**Conclusions:** Our study findings tentatively conclude that paracetamol can induce early patent ductus arteriosus closure without significant side effects but that its efficacy is not superior to that of indomethacin.

## Introduction

Hemodynamically significant patent ductus arteriosus (PDA) is regularly related to morbidity and mortality among premature infants (1, 2). Only 70% of infants born at 1,000–1,500 g and only 30–35% of infants born at < 1,000 g experience spontaneous PDA closure within 7 days of birth (3, 4). Treating PDA to promote rapid ductal closure may be crucial. Owing to the risks associated with surgery, medication is the first-line treatment (5). Indomethacin and ibuprofen, prostaglandin inhibitors that are commonly used to achieve PDA closure (6, 7), act on active cyclooxygenase (COX) receptors to promote ductal constriction by inhibiting prostaglandin synthesis (8). However, these drugs may induce severe adverse effects including isolated perforation, renal impairment, hyperbilirubinemia, and necrotizing enterocolitis (NEC) (6, 9–11). Most of these contraindications are associated with the pharmacological effects produced by ibuprofen or indomethacin, including a decrease in concentration-related prostaglandin synthesis by non-selective inhibition of the COX receptor of the prostaglandin H2 synthetase enzyme (12). Recent studies demonstrated the effectiveness of paracetamol (a prostaglandin synthetase inhibitor) as an alternative therapy for PDA closure in patients with contraindications for indomethacin or ibuprofen or those who have not been successfully treated with these drugs, which has caused great concern among neonatologists (13–16). Paracetamol is believed to work on prostaglandin synthetase in the peroxidase (POX) receptor of the enzyme, boosting paracetamol-mediated inhibition at decreased local peroxide concentrations (17) and immediately inhibiting prostaglandin synthase activity (18). POX is activated when the peroxide concentration is 10 times lower than that of COX (19). This difference may allow POX inhibition to be optimally effective under conditions of low COX inhibitory activity (20). To date, although a number of correlative randomized controlled trials (RCTs) have compared the therapeutic efficacies of paracetamol and other drugs for PDA closure, most achieved insignificant results (21–25). Paracetamol's efficacy and safety for PDA closure in premature or low-birth-weight infants (or both) have not been fully determined.

### Objectives

This systematic review aimed to confirm paracetamol's efficacy and safety compared with those of other drugs or placebo by reviewing RCTs in the literature to increase the sample size.

### Research Question

Is paracetamol effective and safe for PDA closure in premature neonates?

## Methods

### Study Design

This systematic review and meta-analysis was created according to the Cochrane Handbook for Systematic Reviews (Intervention version) and complied with the Preferred Reporting Items for Systematic Reviews and Meta-Analyses (PRISMA) statement (26).

### Participants, Interventions, and Comparators

Samples were <37 weeks' gestation premature infants or <2,500-g low-birth-weight infants with echocardiography-confirmed PDA regardless of postnatal age. Paracetamol was administered to achieve PDA closure.

### Inclusion and Exclusion Criteria

The inclusion criteria for screening studies were as follows: RCTs and quasi-RCTs comparing paracetamol with other drugs or placebo for PDA closure in our target population (regardless of drugs given via oral, intravenous, or rectal route and at any dose). The exclusion criteria were as follows: (i) incomplete article or not published in English (or both); and (ii) administration of paracetamol was not used to achieve PDA closure.

### Types of Outcome Measures

Our outcome types consisted of four primary outcomes and 23 secondary outcomes. Specific outcomes are shown in Table 1.

**Table 1:** Specific outcome measures.

**Primary outcomes**
1. Primary PDA closure (defined as echocardiography confirmed closure of PDA after the first course of the treatment)
2. Overall PDA closure (defined as echocardiography confirmed closure of PDA after one or more courses of the treatment)
3. Neurodevelopmental impairment (NDI) at any age reported (neurodevelopmental outcome assessed by a standardized and validated assessment tool or a child developmental specialist, or both)
4. Moderate-to-severe cerebral palsy at any age reported (neurodevelopmental outcome assessed by a standardized and validated assessment tool or a child developmental specialist, or both)
**Secondary outcomes**
1. All-cause mortality during hospital stay
2. Surgical closure of the PDA
3. Mean days/hours needed for closure of PDA
4. Bronchopulmonary dysplasia (BPD)
5. Pulmonary hemorrhage (blood-stained liquid flowing from the trachea of the infant)
6. Intraventricular hemorrhage (IVH) (all grades)
7. Severe IVH (grades III and IV)
8. Periventricular leukomalacia (PVL)
9. Necrotizing enterocolitis (NEC) (any stage)
10. Gastrointestinal (GI) bleed
11. Retinopathy of prematurity (ROP) (according to the International Classification of ROP); any stage and stage ≥3
12. Oliguria (defined as <1 cm^3^/kg/h) during treatment
13. Sepsis (clinical symptoms and signs of sepsis and a positive blood bacterial culture)
14. Serum or plasma levels of creatinine (mmol/L) after treatment
15. Serum or plasma levels of aspartate transaminase (AST) (IU/L) following treatment
16. Serum or plasma levels of alanine transaminase (ALT) (IU/L) following treatment
17. Serum bilirubin (mmol/L) following treatment
18. Hyperbilirubinemia (serum bilirubin level higher than the exchange level according to the postnatal age and body weight)
19. Duration of hospitalization (total length of hospitalization from birth to discharge home or death) (days)
20. Serum blood urea nitrogen (BUN)
21. Platelet count
22. Serum glutamic-oxaloacetic transaminase level
23. Serum glutamate pyruvate transaminase level

*References: The definition of the outcomes was referred to Yang et al. (23), El-Mashad et al. (24), Ohlsson et al. (27), Huang et al. (28), and Das et al. (29)*.

### Search Strategy

The Cochrane Library, PubMed, CINAHL, and EMBASE databases were searched from the date of their inception to March 2018 to identify published systematic reviews or meta-analyses. Among them, we recognized original RCTs and quasi-RCTs. We also searched the same databases for studies published from December 2013 to March 2018 to identify recently published RCTs and quasi-RCTs. We imposed no language restrictions. The main search terms included “paracetamol,” “ductus arteriosus,” and their synonyms (the specific search strategy used in PubMed is reported in Table 2). We subsequently conducted an updated search (the second search) for studies limited to March 2018 to March 2019 using the same search strategy and searched for terms used in the first search.

**Table 2:** Search strategy for PubMed database.

#1 paracetamol[mh] OR paracetamol OR acetaminophen[mh] OR acetaminophen #2 “Ductus Arteriosus, Patent”[mh] OR “Ductus Arteriosus”[mh] OR Ductus Arteriosus OR “patent ductus arteriosus” OR PDA #3 (“infant, newborn”[mh] OR newborn OR neonate OR neonatal OR premature OR low birth weight OR VLBW OR LBW or infan* or neonat*) NOT (animals [mh] NOT humans [mh]) #4 systematic[sb] OR Meta-Analysis[ptyp] #5 #1 AND #2 AND #3 AND #4 #6 randomized controlled trial [pt] OR controlled clinical trial [pt] OR Clinical Trial[ptyp] OR randomized [tiab] OR placebo [tiab] OR clinical trials as topic [mesh: noexp] OR randomly [tiab] OR trial [ti] #7 #1 AND #2 AND #3 AND #6

### Data Sources, Studies Sections, and Data Extraction

Two researchers independently estimated the study qualification on the basis of pre-established criteria and extracted the relevant information from every included study, as follows: publication year, lead author; country conducting trials; characteristics of participants, method of diagnosis; exposure/intervention (paracetamol or any other drug, dose of the drugs, trial duration, and number of courses), and data of results (outcome measures, effect, significance, and adverse events). If studies had more than two sets or allowed multiple tests, we obtained only the requisite data and information reported. Differences were resolved through negotiation or third-party intervention.

### Assessment of Risk of Bias

Two researchers independently evaluated the selected trials by applying the criteria listed in the Cochrane Handbook and rated these trials as being of low, high, or unclear risk (30). Differences were resolved as described above.

### Data Analysis

We executed a meta-analysis using the Mantel–Haenszel or inverse variance statistical method to calculate risk ratios (RRs) or mean difference (MD) and 95% confidence intervals (CIs). We used Cochran's *Q*-test to assess heterogeneity and values of *P* < 0.10 were considered significantly heterogenous (31). Based on the Cochrane Handbook, when there was minimal evidence of heterogeneity, a fixed-effects meta-analysis model was used. When the effect-estimated *I*^2^ value was >30%, the random-effects model was used, and we would attempt to determine the reason for the heterogeneity. The sensitivity analysis was performed by stratified analysis. Given that few studies were included in the secondary outcomes part of the study, the subgroup analysis included only the primary outcomes. Subgroups were pre-specified according to administration route (oral, intravenous, or other), gestational age (including <28, 28–32, and 33–36 weeks) and birth weight (including <1,000, 1,000–1,500, and 1,501–2,500 g), which allowed us to estimate whether the relationship between paracetamol and other drugs or placebo was changed by the participants' characteristics. We intended to evaluate potential publication bias by examining a funnel plot. Two-tailed *P* < 0.05 were considered statistically significant. RevMan version 5.3 was used for all of the analyses.

## Results

### Description of Studies

**Figure 1:**
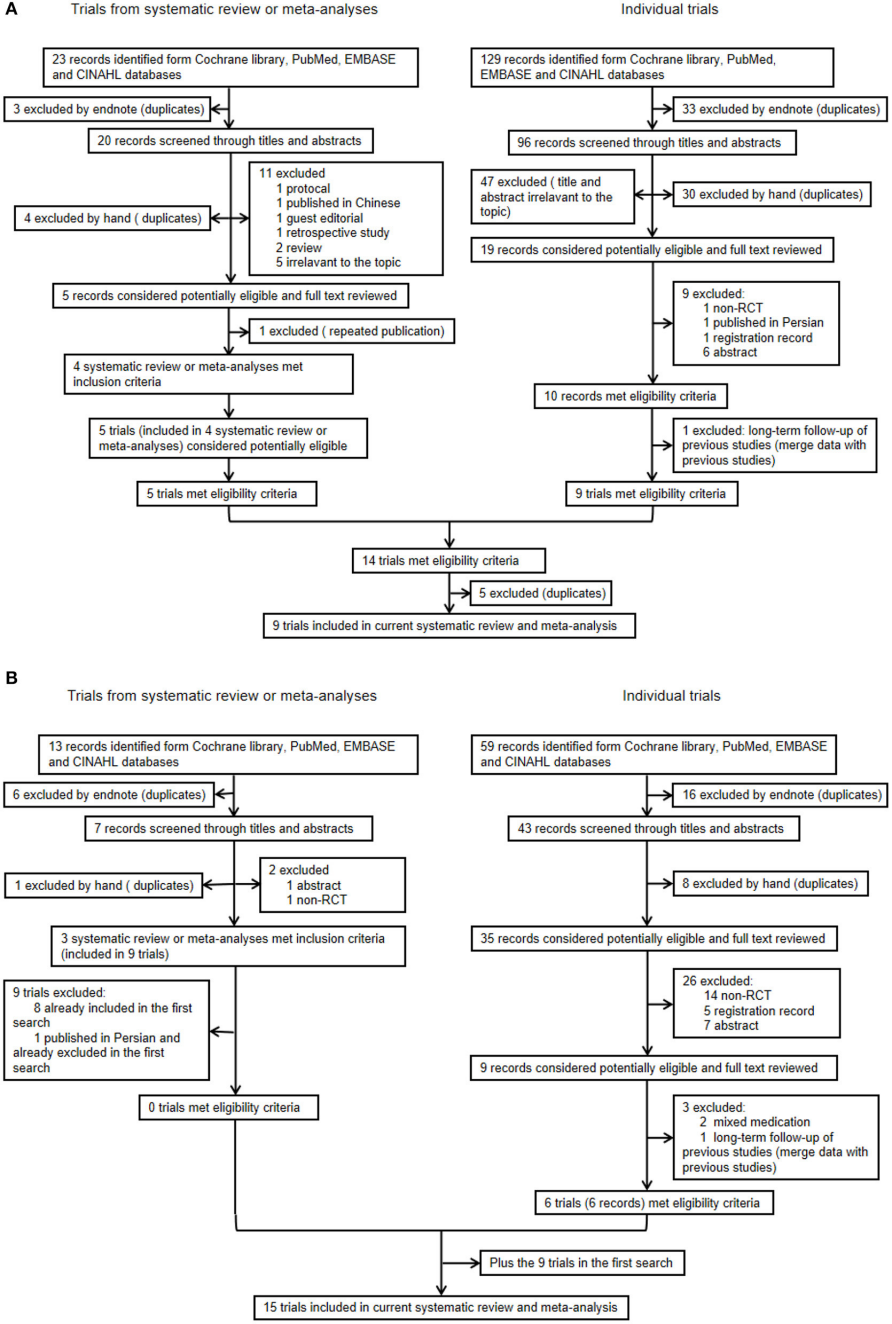
**(A)** Flow chart (first search) and **(B)** Flow chart (second search).

In the first search, of the 23 systematic reviews and 129 citations retrieved, four systematic reviews (27–29, 32) were assessed to extract RCTs or quasi-RCTs (Table 3). The full texts of 10 articles (2, 13, 14, 21–25, 36, 37) that met the inclusion criteria were assessed for eligibility after retrieval of the RCTs or quasi-RCTs (Figure 1). Of these, two (2, 22) came from the same study: one reported short-term outcomes, whereas the other reported long-term outcomes. Thus, the extracted data from those two articles were considered those of a single study. Therefore, nine trials were included in this systematic review in the first search. In the second search, of 13 systematic reviews and 59 citations retrieved, three systematic reviews (33–35) were assessed to extract RCTs or quasi-RCTs (Table 3). Seven records (38–44) containing six trials met our inclusion criteria. Therefore, by summarizing the trials of the first and second searches, 15 trials were eventually included in our review (Table 4). The primary characteristics of the selected trials are displayed in Table 4. The included outcomes of the selected studies are reported in Table 5.

**Table 3:** Randomized trials included in systematic reviews or meta-analyses evaluating paracetamol for patent ductus arteriosus.

**References**	**Systematic reviews or meta-analyses**						
	**Das et al. (29)**	**Ohlsson et al. (27)**	**Terrin et al. (32)**	**Huang et al. (28)**	**Hossain et al. (33)**	**Mitra et al. (34)**	**Ohlsson et al. (35)**
Dang et al. (21)	Y	Y	Y	Y	Y	Y	Y
Oncel et al. (22)	Y	Y	Y	Y	Y	Y	Y
Dash et al. (36)					Y	Y	Y
Bagheri et al. (13)				Y	Y	Y	
Yang et al. (23)				Y	Y	Y	Y
Harkin et al. (37)					Y		Y
El-Mashad et al. (24)				Y	Y		Y
Al-Lawama et al. (25)							Y

*Y, yes (each “Y” indicates that this trial was included in the systematic reviews or meta-analyses of the corresponding column)*.

**Table 4:** Characteristics of included studies.

**References**	**BW (g)**	**GA (weeks)**	**Location**	**Sample size**	**PA (days)**	**Ductal diameter (mm)**	**Intervention**	**Route**	**Dose (mg/kg/day)**	**Duration (days)**	**Timing**
Al-Lawama et al. (25)	1,126	28.0	Jordan	22	≤ 5	NR	Paracetamol/ibuprofen	Oral/oral	40–40–40/10–10–10	3	First-line therapy
Asadpour et al. (38)	<1,750	<37	Iran	50	NR	3.7	Paracetamol/ibuprofen	Oral/oral	10–10–10/10–5–5	3	First-line therapy
Babaei et al. (39)	1,959.4	31.67	Iran	69	4.84	2.3	Paracetamol/no intervention	Oral	15–15–15/0–0–0	3	First-line therapy
Bagheri et al. (13)	1,644	31.6	Iran	150	3	NR	Paracetamol/ibuprofen	Oral/oral	60–60–60/20–10–10	3	First-line therapy
Balachander et al. (40)	1,524.1	31.56	India	110	NR	2.39	Paracetamol/ibuprofen	Oral/oral	15–15–15/10–5–5	3	First-line therapy
Dang et al. (21)	1,562	31.1	China	160	≤ 14	2.4	Paracetamol/ibuprofen	Oral/oral	60–60–60/10–5–5	3	First-line therapy
Dani et al. (14)	976	27.8	Italy	21	4	1.8	Paracetamol/ibuprofen	IV/IV	60–60–60/10–5–5	3	First-line therapy
Dash et al. (36)	1,008	28.7	India	77	<1	2.1	Paracetamol/indomethacin	Enteral/IV	60–60–60/14.4–14.4–14.4	7	First-line therapy
El-Farrash et al. (41)	1,635	31.13	India	60	6.95	2.34	Paracetamol/ibuprofen	Oral/oral	15–15–15/10–5–5	3	First-line therapy
El-Mashad et al. (24)	1,067	25.7	Egypt	300	3	2.7	Paracetamol/ibuprofen/ indomethacin	IV/IV	60–60–60/10–5–5/0.4–0.4–0.4	3/3/1.5	First-line therapy
Harkin et al. (37)	1,170	28.4	Finland	63	<1	1.5	Paracetamol/placebo	IV/IV	42.5–30–30–30/42.5–30–30–30	4	First-line therapy
Hochwald et al. (42)	645.5	27.45	Israel	24	6.45	3.35	Paracetamol + ibuprofen/ ibuprofen + placebo	IV/IV	(10–5.5) + (20–10–10)/(10–5.5) + (20–10–10)	3	First-line therapy
Kluckow et al. (44)	994.5	27.05	Australia	55	25	2.5	Paracetamol/placebo	Oral/oral	25–15–15–15–15/25–15–15–15–15	5	First-line therapy
Oncel et al. (22)	952	27.3	Turkey	80	2-4	2.3	Paracetamol/ibuprofen	Oral/oral	60–60–60/10–5–5	3	First-line therapy
Yang et al. (23)	2,155	33.5	China	87	6	1.965	Paracetamol/ibuprofen	Oral/oral	60–60–60/10–5–5	3	First-line therapy

*BW, birth weight; GA, gestational age; PA, postnatal age*.

**Table 5:** Included outcomes of included studies.

**References**	**Paracetamol vs. ibuprofen**	**Paracetamol vs. indomethacin**	**Paracetamol vs. placebo**
Al-Lawama et al. (25)	O1, O2, O5, O8, O9, O10, O13, O17		
Asadpour et al. (38)	O2, O14, O18, O19, O20, O21, O24		
Babaei et al. (39)			O1, O2
Bagheri et al. (13)	O1, O2, O5, O10, O11, O15, O17,		
Balachander et al. (40)	OO1, O2, O8, O13, O15, O10, O23,		
Dang et al. (21)	O1, O2, O5, O7, O8, O10, O11, O12, O13, O14, O15, O16, O17, O18, O22		
Dani et al. (14)	O1, O2, O8, O10, O11, O13, O17, O23		
Dash et al. (36)		O1, O2, O5, O8, O9, O10, O12, O13, O14, O15, O17	
El-Farrash et al. (41)	O1, O2, O5, O6, O8, O18, O19, O20, O21, O23, O24,		
El-Mashad et al. (24)	O1, O2, O6, O9, O10, O13, O14, O15, O17, O18, O21, O24, O25, O26, O27	O1, O2, O6, O9, O10, O13, O14, O15, O17, O18, O21, O24, O25, O26, O27	
Harkin et al. (37)			O1, O2, O5, O7, O8, O10, O11, O13, O15, O16, O17
Hochwald et al. (42)			O1, O2, O8, O10, O11, O12, O13, O15, O16, O17, O19, O20
Juujärvi et al. (43)			O4
Kluckow et al. (44)			O1, O6, O8, O9, O13, O14, O15, O23
Oncel et al. (2)	O3, O4,		
Oncel et al. (22)	O1, O2, O5, O6, O9, O13, O14, O15, O17, O18, O19, O20, O21, O23, O24		
Yang et al. (23)	O1, O2, O8, O10, O13, O16, O18, O25, O27		

*O, outcomes; O1, primary PDA closure; O2, overall PDA closure; O3, neurodevelopmental impairment (NDI); O4, moderate-to-severe cerebral palsy; O5, all-cause mortality during hospital stay; O6, surgical closure of the PDA; O7, mean days/hours needed for closure of PDA; O8, BPD (bronchopulmonary dysplasia); O9, pulmonary hemorrhage; O10, IVH (intraventricular hemorrhage); O11, severe IVH (grades III and IV); O12, PVL (periventricular leukomalacia); O13, NEC (necrotizing enterocolitis); O14, gastrointestinal (GI) bleed; O15, ROP (retinopathy of prematurity); O16, oliguria; O17, sepsis; O18, serum creatinine—serum or plasma levels of creatinine (mmol/L) after treatment; O19, serum or plasma levels of aspartate transaminase (AST) (IU/L) following treatment; O20, serum or plasma levels of alanine transaminase (ALT) (IU/L) following treatment; O21, serum bilirubin (mmol/L) following treatment; O22, hyperbilirubinemia; O23, duration of hospitalization; O24, serum blood urea nitrogen (BUN); O25, platelet count; O26, serum SGOT level—serum glutamic-oxaloacetic transaminase level; O27, serum SGPT level—serum glutamate pyruvate transaminase level*.

### Risk of Bias

**Figure 2:**
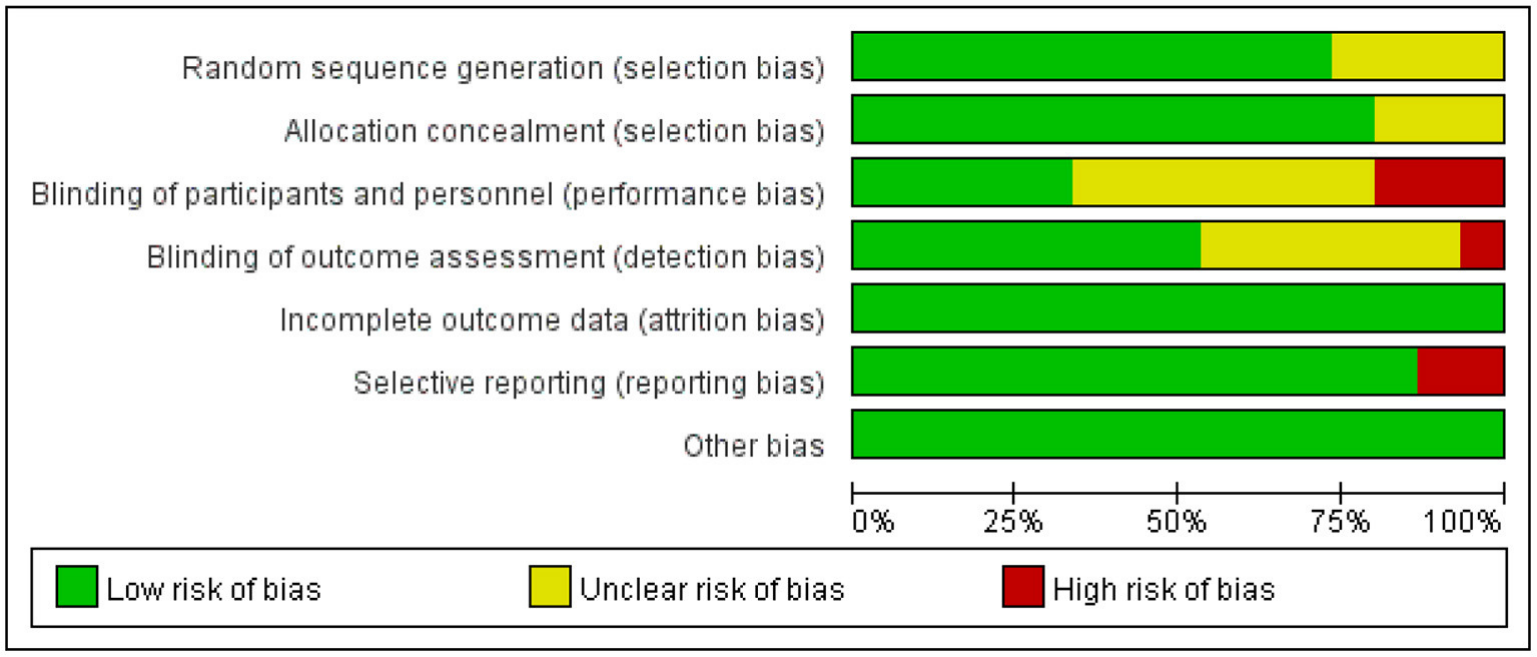
Assessment of risk of bias in randomized controlled trials.

**Figure 3:**
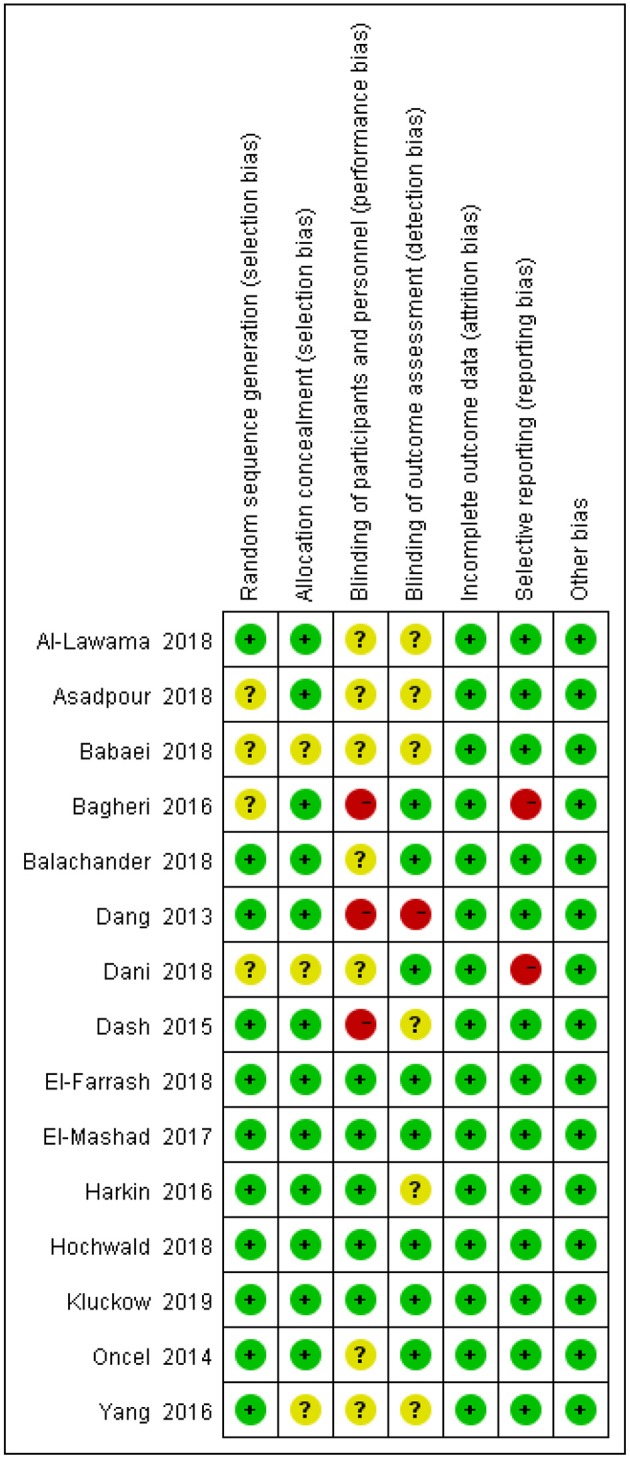
Results of the risk of bias.

The degrees of bias of the selected trials were low to high because of their double-blind, single-blind, or open-label designs. Figures 2, 3 display the evaluation of the degrees of bias.

### Effect of Interventions

#### Paracetamol vs. Ibuprofen

**Figure 4:**
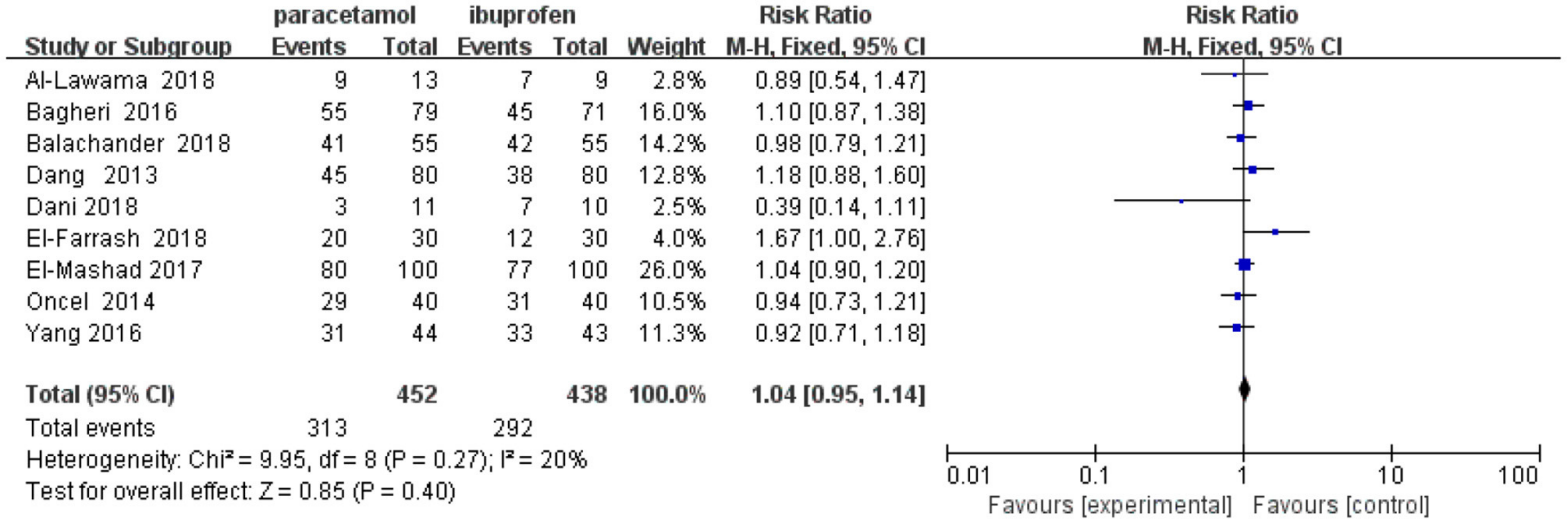
Primary PDA closure.

When paracetamol was compared with ibuprofen, all 27 outcomes were reported (Table 5). The results of the study showed no significant differences in the pooled results of the primary outcomes between the two set comparison groups regardless of whether a subgroup analysis was performed (Table 6 and Figure 4 show the forest plot of the meta-analysis focusing on primary PDA closure). Among the secondary outcomes, only the pooled results of three outcomes showed statistically significant intergroup differences. Specifically, compared with the ibuprofen group, in the paracetamol group, the mean number of hours needed for PDA closure was significantly shorter [MD, −11.76 (95% CI, −12.88 to −10.64), *P* < 0.001] and the proportion of gastrointestinal (GI) bleeding [RR, 0.19 95% CI, 0.07–0.56), *P* = 0.002] and hyperbilirubinemia [RR, 0.57 (95% CI, 0.34–0.97), *P* = 0.04] were significantly reduced. There was no heterogeneity in these comparisons (Table 5).

**Table 6:** The pooled results of meta-analyses.

**Interventions**	**Outcomes**	**Subgroups**	**Trials (no.)**	**RR/MD (95% CI)**	***P*_**RR/MD**_**	***I*^**2**^ (%)**	***P*_**Het**_**
**Paracetamol vs. ibuprofen**	**Primary outcomes**						
	Primary PDA closure		9	1.04 (0.95, 1.14)	0.40	20	0.27
		Oral	7	1.06 (0.95, 1.18)	0.28	13	0.33
		IV	2	0.73 (0.28, 1.90)	0.51	72	0.06
		Gestational age <28 weeks	3	0.95 (0.76, 1.20)	0.69	48	0.15
		Gestational age ≥28 weeks	6	1.08 (0.96, 1.22)	0.19	20	0.28
		Birth weight <1,000 g	2	0.69 (0.29, 1.64)	0.40	65	0.09
		Birth weight: 1,000 to 1,500 g	2	1.02 (0.89, 1.18)	0.73	0	0.56
		Birth weight: 1,501 to 2,500 g	5	1.09 (0.97, 1.23)	0.16	32	0.21
	Overall PDA closure		10	1.01 (0.95, 1.07)	0.82	0	0.77
		Oral	8	1.00 (0.93, 1.07)	0.95	0	0.76
		IV	2	1.04 (0.92, 1.16)	0.54	6	0.30
		Gestational age <28 weeks	3	1.03 (0.94, 1.12)	0.59	0	0.53
		Gestational age ≥28 weeks	7	1.00 (0.93, 1.07)	0.95	0	0.66
		Birth weight <1,000 g	2	0.96 (0.83, 1.12)	0.61	0	0.39
		Birth weight: 1,000 to 1,500 g	2	1.05 (0.94, 1.16)	0.40	0	0.35
		Birth weight: 1,501 to 2,500 g	6	1.00 (0.93, 1.08)	1.00	0	0.57
	NDI		1	0.93 (0.44, 1.96)	0.85	/	/
	Moderate-to-severe cerebral palsy		1	2.07 (0.41, 10.46)	0.38	/	/
	**Secondary outcomes**						
	All-cause mortality during hospital stay		5	1.16 (0.68, 1.97)	0.60	11	0.34
	Surgical closure of the PDA		3	0.63 (0.33, 1.19)	0.15	0	0.70
	Mean hours needed for closure of PDA		1	−11.76 (−12.88, −10.64)	<0.001	/	/
	Bronchopulmonary dysplasia (BPD)		6	0.80 (0.41, 1.55)	0.51	0	0.81
	Pulmonary hemorrhage		3	0.47 (0.14, 1.53)	0.21	0	0.94
	Intraventricular hemorrhage (IVH) (all grades)		7	1.04 (0.65, 1.67)	0.86	0	0.82
	Severe IVH (grades III and IV)		3	1.13 (0.37, 3.42)	0.83	0	0.48
	Periventricular leukomalacia (PVL)		1	1.20 (0.38, 3.77)	0.76	/	/
	Necrotizing enterocolitis (NEC) (any stage)		7	1.03 (0.66, 1.60)	0.90	0	0.84
	Gastrointestinal bleed (GI)		4	0.19 (0.07, 0.56)	0.002	0	0.90
	Retinopathy of prematurity (ROP)		5	0.85 (0.59, 1.23)	0.39	0	0.63
	Decreased urine output (defined as <1 cm^3^/kg/h) during treatment		2	0.44 (0.12, 1.59)	0.21	33	0.22
	Sepsis		6	0.96 (0.70, 1.31)	0.79	0	0.63
	Serum or plasma levels of creatinine (mmol/L) after treatment		6	−5.29 (−11.56, 0.98)	0.10	80	0.0001
	Serum or plasma levels of aspartate transaminase (AST) (IU/L) following treatment		3	−0.19 (−5.90, 5.53)	0.95	65	0.06
	Serum or plasma levels of alanine transaminase (ALT) (IU/L) following treatment		3	0.77 (−2.40, 3.93)	0.63	58	0.09
	Serum bilirubin (mmol/L) following treatment		4	0.17 (−0.74, 1.09)	0.71	82	0.0007
	Hyperbilirubinemia		1	0.57 (0.34, 0.97)	0.04	/	/
	Duration of hospitalization		4	−0.95 (−8.30, 6.40)	0.80	44	0.15
	Serum blood urea nitrogen (BUN)		4	−0.59 (−2.52, 1.35)	0.55	66	0.03
	Platelet count		2	7.36 (−60.54, 75.25)	0.83	92	0.0005
	Serum glutamic-oxaloacetic transaminase (SGOT) level		1	−0.50 (−4.15, 3.15)	0.79	/	/
	Serum glutamate pyruvate transaminase (SGPT) level		2	0.35 (−0.63, 1.33)	0.49	0	0.83
**Paracetamol vs. indomethacin**	**Primary outcomes**						
	Primary PDA closure		2	1.01 (0.91, 1.12)	0.88	0	0.45
	Overall PDA closure		2	1.02 (0.94, 1.11)	0.58	0	0.61
	NDI		0	/	/	/	/
	Moderate-to-severe cerebral palsy		0	/	/	/	/
	**Secondary outcomes**						
	All-cause mortality during hospital stay		1	1.03 (0.43, 2.46)	0.95	/	/
	Surgical closure of the PDA		1	0.92 (0.44, 1.92)	0.83	/	/
	Mean days needed for closure of PDA		0	/	/	/	/
	Bronchopulmonary dysplasia (BPD)		1	0.78 (0.45, 1.38)	0.40	/	/
	Pulmonary hemorrhage		2	1.12 (0.05, 26.99)	0.94	73	0.05
	Intraventricular hemorrhage (IVH) (all grades)		2	0.79 (0.34, 1.84)	0.59	32	0.22
	Severe IVH (grades III and IV)		0	/	/	/	/
	Periventricular leukomalacia (PVL)		1	1.17 (0.47, 2.92)	0.73	/	/
	Necrotizing enterocolitis (NEC) (any stage)		2	0.39 (0.14, 1.06)	0.06	0	0.68
	Gastrointestinal bleed (GI)		2	0.44 (0.03, 7.49)	0.57	85	0.01
	Retinopathy of prematurity (ROP)		2	0.73 (0.35, 1.54)	0.41	65	0.09
	Decreased urine output (defined as <1 cm^3^/kg/h) during treatment		0	/	/	/	/
	Sepsis		2	1.18 (0.80, 1.74)	0.41	0	0.68
	Serum or plasma levels of creatinine (mmol/L) after treatment		1	−30.94 (−34.34, −27.54)	<0.001	/	/
	Serum or plasma levels of aspartate transaminase (AST) (IU/L) following treatment		0	/	/	/	/
	Serum or plasma levels of alanine transaminase (ALT) (IU/L) following treatment		0	/	/	/	/
	Serum bilirubin (mmol/L) following treatment		1	0.06 (0.01, 0.11)	0.03	/	/
	Hyperbilirubinemia		0	/	/	/	/
	Duration of hospitalization		0	/	/	/	/
	Serum blood urea nitrogen (BUN)		1	−11.40 (−12.30, −10.50)	<0.001	/	/
	Platelet count		1	112.00 (103.02, 120.98)	<0.001	/	/
	Serum glutamic-oxaloacetic transaminase (SGOT) level		1	0.00 (−3.76, 3.76)	1.00	/	/
	Serum glutamate pyruvate transaminase (SGPT) level		1	0.60 (−0.46, 1.66)	0.27	/	/
**Paracetamol vs. placebo**	Primary PDA closure		4	2.62 (0.90, 7.57)	0.08	80	0.002
		Oral	2	5.33 (2.39, 11.86)	<0.001	0	0.68
		IV	2	1.41 (0.99, 2.01)	0.06	0	0.44
		Gestational age <28 weeks	2	3.03 (1.05, 8.76)	0.04	9	0.29
		Gestational age ≥28 weeks	2	2.44 (0.49, 12.21)	0.28	92	0.0003
		Birth weight <1,000 g	2	3.03 (1.05, 8.76)	0.04	9	0.29
		Birth weight: 1,000 to 1,500 g	1	1.29 (0.91, 1.83)	0.15	/	/
		Birth weight: 1,501 to 2,500 g	1	4.95 (2.16, 11.34)	0.0002	/	/
	Overall PDA closure		3	2.22 (0.45, 10.97)	0.33	96	<0.001
		Oral	1	6.23 (2.77, 14.03)	<0.001	/	/
		IV	2	1.29 (0.56, 2.96)	0.55	80	0.03
		Gestational age <28 weeks	1	2.00 (0.98, 4.09)	0.06	/	/
		Gestational age ≥28 weeks	2	2.38 (0.13, 42.02)	0.55	98	<0.001
		Birth weight <1,000 g	1	2.00 (0.98, 4.09)	0.06	/	/
		Birth weight: 1,000–1,500 g	1	0.94 (0.74, 1.19)	0.60	/	/
		Birth weight: 1,501–2,500 g	1	6.23 (2.77, 14.03)	<0.001	/	/
	Moderate-to-severe cerebral palsy		1	0.36 (0.02, 8.45)	0.53	/	/
	**Secondary outcomes**						
	All-cause mortality during hospital stay		1	0.36 (0.02, 8.45)	0.53	/	/
	Surgical closure of the PDA		1	3.11 (0.13, 73.11)	0.48	/	/
	Pulmonary hemorrhage		1	2.07 (0.20, 21.56)	0.54	/	/
	Bronchopulmonary dysplasia (BPD)		3	0.76 (0.52, 1.11)	0.16	0	0.37
	Sepsis		2	1.04 (0.41, 2.63)	0.93	0	0.49
	Necrotizing enterocolitis (NEC) (any stage)		3	0.52 (0.10, 2.74)	0.44	0	0.83
	Retinopathy of prematurity (ROP)		3	1.22 (0.43, 3.42)	0.71	11	0.32
	Intraventricular hemorrhage (IVH) (all grades)		2	0.70 (0.37, 1.35)	0.29	0	0.76
	Severe IVH (grades III and IV)		2	0.82 (0.26, 2.58)	0.73	0	0.81
	Periventricular leukomalacia (PVL)		1	0.33 (0.01, 7.45)	0.49	/	/
	Decreased urine output (defined as <1 cm^3^/kg/h) during treatment		2	0.71 (0.29, 1.78)	0.47	0	0.73
	Gastrointestinal bleed (GI)		1	1.04 (0.07, 15.76)	0.98	/	/
	Mean hours needed for closure of PDA		1	−219.0 (−464.26, 26.26)	0.08	/	/
	Duration of hospitalization		1	Lack of data	>0.05	/	/

*IV, intravenous injection; P_Het_, P-value of heterogeneity test*.

#### Paracetamol vs. Indomethacin

When paracetamol was compared with indomethacin, 18 outcomes were reported, including two primary outcomes and 16 secondary outcomes (Table 5). The results showed no significant intergroup differences in the pooled results of primary outcomes (Table 6). Among the secondary outcomes, only the pooled results of the four outcomes were statistically different between the two groups. However, the reports of these four outcomes were all from the same study (24). Specifically, vs. those in paracetamol group, serum creatinine level [MD, −30.94 (95% CI, 34.34–27.54), *P* < 0.001] and blood urea nitrogen level [MD, −11.40 (95% CI, −12.30 to −10.50), *P* < 0.001] were significantly increased in the indomethacin group (*P* < 0.001), whereas platelet count [MD, 112.00 (95% CI, 103.02–120.98), *P* < 0.001] and serum bilirubin level after treatment [MD, 0.06 (95% CI, 0.01–0.11), *P* = 0.03] were significantly lower (*P* < 0.001) in the indomethacin group (Table 6).

#### Paracetamol vs. Placebo

Seventeen outcomes were reported in the comparison of paracetamol and placebo (Table 5). Four trials reported the effects of paracetamol vs. placebo for PDA closure. Specifically, two compared paracetamol and placebo, one compared paracetamol and no intervention, and one compared paracetamol plus ibuprofen vs. ibuprofen plus placebo. Although the last comparison was a combined therapy, the study design was a prospective, randomized, double-blind, placebo-controlled pilot study. So in addition to the influence of paracetamol, the latter two comparisons were similar to those between paracetamol and placebo after balancing differences between groups and were therefore classified as paracetamol vs. placebo. Our meta-analysis showed that the oral paracetamol group better promoted primary PDA closure than did the placebo group. In addition, in the gestational age <28 weeks, body weight <1,000 g, and body weight of 1,501–2,500 g, the paracetamol group better promotes primary PDA closure. Regarding overall PDA closure, the oral paracetamol group, compared with the placebo group, promoted PDA closure for infants weighing <1,000 g and those weighing 1,501–2,500 g. According to the results, no significant intergroup differences existed between paracetamol and placebo in other outcomes (Table 6).

#### Publication Bias

**Figure 5:**
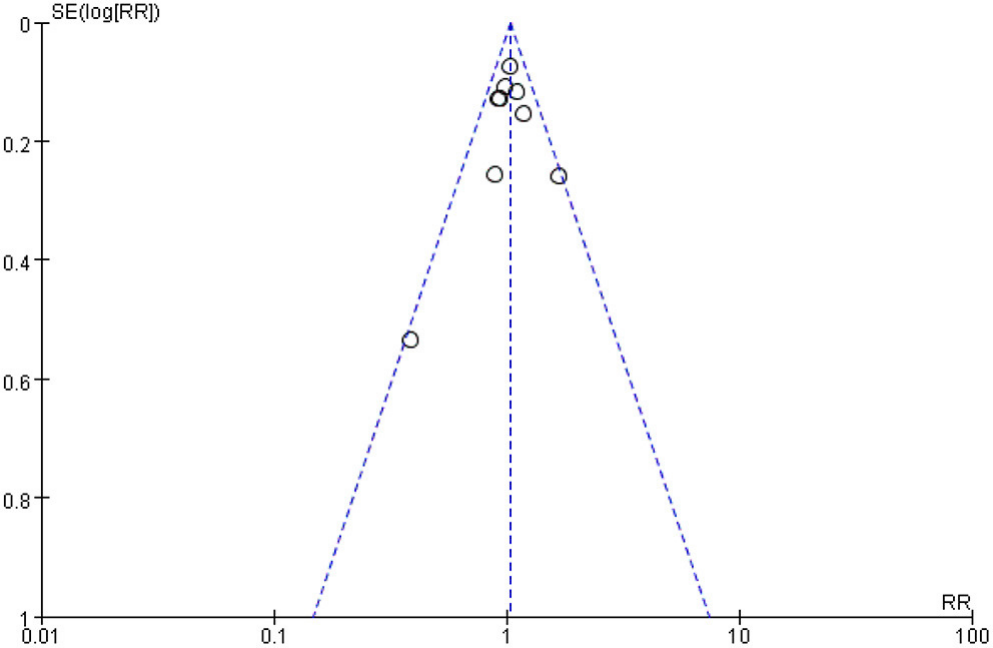
Funnel Plot of the comparing primary PDA closure of paracetamol and ibuprofen on PDA closure in preterm or low birth weight (or both) infants.

We inspected a funnel plot for the comparison of primary PDA closure of paracetamol and ibuprofen in our target population and found almost no publication bias (Figure 5).

## Discussion

This systematic review meta-analyzed the use of paracetamol for PDA closure in premature infants. Our findings can enhance our understanding of the theme.

The following outcomes were not used in the subgroup analyses: mean days needed for PDA closure, GI bleeding, and hyperbilirubinemia. Our study results showed no significant difference between the paracetamol and ibuprofen groups internal or external of the subgroup analyses. Compared with the ibuprofen group, the paracetamol group had shorter mean days for PDA closure, a lower risk of GI bleeding, and lower risk of hyperbilirubinemia. A recent Cochrane systematic review demonstrated no difference in efficacy between oral paracetamol and oral ibuprofen (35). As the same two RCTs were included, Das et al. and Terrin et al. reported the same conclusion as the aforementioned Cochrane systematic review (29, 32). Huang et al. stated that no significant difference existed between paracetamol and ibuprofen in PDA closure in premature neonates by summarizing the results of five RCTs, but the paracetamol group, compared with the ibuprofen group, had a reduced risk of renal failure as well as GI bleeding (28).

In our research, the comparison of paracetamol and ibuprofen identified nine studies that reported primary closure and 10 studies that reported total closure. The results showed that, consistent with other studies (9, 24), paracetamol was as efficacious as ibuprofen in accelerating PDA closure in premature infants. The primary closure and overall closure rates after paracetamol therapy (313/452 = 69.25%; 398/477 = 83.44%) were more or less similar to those after ibuprofen therapy (292/438 = 66.67%; 384/463 = 82.94%). The overall closure rate of our study was slightly higher than that reported by El-Mashad et al. (24), probably because of the higher weights of the infants with a higher mean gestational age after the merger. Higher prostaglandin receptor expression in the PDA wall demonstrated a lesser response to COX inhibition in young premature infants (45). In addition, a longer average treatment time after the merger may have led to a higher closure rate. Consistent with previous studies, our research indicated that the ibuprofen group had a higher incidence of GI bleeding. The potential peripheral effect of vasoconstriction and the potential antiplatelet aggregation effect of ibuprofen could explain the higher GI bleeding tendency in the ibuprofen group (46, 47), and paracetamol did not harm the GI mucosa (48). Another study showed that paracetamol was recommended for infants with clinical contraindications to non-steroidal anti-inflammatory drugs (9). Because only one study reported the mean hours needed for PDA closure, our results supported the conclusion of Das et al. (29) in that the paracetamol group required less time for closure than did the ibuprofen group. Only one study reported hyperbilirubinemia (49). A higher risk of hyperbilirubinemia with ibuprofen use may be explained by the ibuprofen albumin binding with consequent bilirubin displacement (49).

Regarding paracetamol treatment of PDA, two studies reported neurodevelopmental outcomes. A subsequent study by Oncel et al. compared the effects of paracetamol and ibuprofen on pharmacological closure and neurodevelopmental outcomes in premature infants between 18 and 24 months of corrected age (2). Juujärvi et al. conducted a follow-up study of the Harkin study and reported the effects of early intravenous paracetamol on pharmacological closure of neurodevelopmental outcomes at corrected age of 2 years (43). Their results showed no difference in neurodevelopmental outcomes in premature infants receiving paracetamol or ibuprofen/placebo. However, paracetamol works on the endocannabinoid system, which refers to brain development (50). Posadas et al. found that paracetamol caused direct toxicity in rat cortical neurons *in vitro* as well as *in vivo*, resulting in apoptosis of the rat cortical neurons (51). In addition, Viberg et al. reported that the effects of neonatal paracetamol exposure on brain development appeared as adult behavior and caused cognitive deficits; likewise, they also changed in response to paracetamol (50). Therefore, rigorous RCTs and cohort studies are needed to clarify the effects of paracetamol on the neurodevelopmental outcomes of infants.

Although some articles reported that paracetamol was safer than indomethacin in terms of side effects (24, 34, 52), our results did not directly produce such results. The main reason was that fewer studies were included. Only one study reported statistically significant adverse outcomes. However, these adverse outcomes did not all point in the same direction (both beneficial and harmful). However, there was a slight trend in the favoring paracetamol over indomethacin in terms of primary PDA closure [RR, 1.01 (95% CI, 0.91–1.12), *P* = 0.88] and overall PDA closure [RR, 1.02 (95% CI, 0.94–1.11), *P* = 0.88] (Table 6), but the difference did not reach statistical significance. This research only included two trials with a lower sample size, which may have led to the current analyses lacking statistical power to support this association. In the USA, many centers use indomethacin as a drug to prevent (severe) intraventricular hemorrhage (IVH) (53), although this is not a property of paracetamol. A study has shown that prophylactic indomethacin administration given in extremely premature infants at level 4 neonatal intensive care units (NICUs) could improve survival but had no significant effect on the incidence of severe IVH or PDA closure (54). Therefore, the current evidences make it difficult to distinguish which of the two drugs is the best.

Regarding the comparison of paracetamol and placebo for PDA closure, four trials satisfied the inclusion criteria. According to our meta-analysis, oral paracetamol achieved more PDA closures, whether primary or total. Paracetamol was also better than placebo for PDA closure in infants at <28 weeks' gestational age, a birth weight <1,000 g, or a birth weight of 1,501–2,500 g. In addition, no significant difference was found between paracetamol and indomethacin in the aspects of all secondary outcomes. Given these results, we tentatively conclude that paracetamol can induce early PDA closure without noticeable side effects. However, because many of the adverse outcomes (such as periventricular leukomalacia and GI bleeding) were reported in only one study, these findings should be treated cautiously owing to the insufficient numbers of patients to thoroughly assess efficiency and safety.

### Limitations

The results of this analysis have several disadvantages. First, we discovered RCTs from published systematic reviews and meta-analyses in English, possibly omitting trials published in other languages that satisfied the inclusion criteria. Second, the included trials had open-label or single-blinded or double-blinded designs, which were not always of high quality. Third, because PDA has a high spontaneous closure rate, it is not a major problem for larger infants. Fourth, the diagnosis and treatment of PDA remain controversial, and the included studies may have had different echocardiographic criteria that may have impacted the outcomes. Fifth, we conducted stratified analyses on the basis of the different characteristics of the premature infants, but owing to the small number of studies, it was difficult to conduct a more detailed analysis. At the same time, the stratification further led to a decrease in sample size, making it difficult to draw accurate conclusions. Finally, in this updated systematic review, because of the few numbers of studies involving paracetamol vs. placebo, we classified the use of ibuprofen + paracetamol vs. ibuprofen + placebo as paracetamol vs. placebo. This comparison was a prospective, randomized, double-blind, placebo-controlled pilot study; in addition to the influence of paracetamol, the comparison was similar to paracetamol vs. placebo after balancing differences between groups, but the combined therapy may have affected the primary or secondary outcomes.

### Further Areas of Research

Double-blind parallel trials and cohort studies of a larger sample should be conducted to further confirm the long- and short-term efficiency and safety of the above drugs and the differences among them. Trials should report all the useful and important outcomes described in this review at a minimum. When using any drug, safety and efficacy should also be studied in different subgroups of premature infants (characteristics that affect therapeutic efficacy include gestational age, birth weight, dosages, administration route, and timing). To reduce the impact of spontaneous closure, trials should select extremely premature infants (gestational age ≤ 24 weeks or birth weight <1,000 g).

## Conclusion

Compared with ibuprofen, paracetamol showed specific effects for PDA closure owing to fewer adverse reactions. Specifically, paracetamol showed shorter mean days needed for closure, a lower percentage of GI bleeding, and lower risk of hyperbilirubinemia. Compared with indomethacin, paracetamol did not differ in efficacy or safety. Compared with placebo, paracetamol could promote PDA closure without adverse reactions in this meta-analysis. These findings tentatively conclude that paracetamol can induce early PDA closure without noticeable side effects but do not demonstrate that paracetamol is superior to indomethacin. Therefore, more well-designed studies are needed to enrich the evidence of this treatment. Finally, because of the controversy in the diagnosis and treatment of PDA in premature infants, this updated systematic review and meta-analysis only summarizes the existing evidence and does not make any recommendations.

## Data Availability Statement

All datasets generated for this study are included in the article/supplementary material.

## Author Contributions

YX, HL, and XJ conceived this study. YX and RH designed the search strategy and screened studies for eligibility. YX, QY, and XJ assessed study risk of bias and the quality of evidence. YX, RH, and QY wrote the first draft of the manuscript and conducted data analysis. HL, MZ, and XJ interpreted the data analysis and critically revised the manuscript.

### Conflict of Interest

The authors declare that the research was conducted in the absence of any commercial or financial relationships that could be construed as a potential conflict of interest.
